# Artroplastia total do joelho com plataforma móvel *versus* plataforma fixa: Uma análise comparativa dos desfechos clínicos de longo prazo e de sobrevida do implante

**DOI:** 10.1055/s-0045-1814105

**Published:** 2025-12-22

**Authors:** Pablo Agustín Ramos Guarderas, Pablo David Ramos Murillo, Carlos Patricio Peñaherrera Carrillo, Francisco Endara Urresta, Daniel Alejandro Ramos Murillo, Alejandro Xavier Barros Castro

**Affiliations:** 1 Clínica Arthros, Quito, Ecuador; 2 Unidade de Medicina do Esporte, Sport Center, Centro Médico-Quirúrgico Olympia, Grupo Quirónsalud, Madrid, Spain; 3 Instituto Nacional de Rehabilitación Luis Guillermo Ibarra Ibarra, Universidad Autónoma de México, Ciudad de México , Mexico; 4 Especialização em Ortopedia e Traumatologia, Faculdade de Medicina, Universidad El Bosque, Bogotá, Colombia; 5 Especialização em Ortopedia e Traumatologia, Escola de Medicina, Universidad Internacional del Ecuador, Quito, Ecuador

**Keywords:** articulação do joelho, artroplastia do joelho, desenho de prótese, prótese do joelho, arthroplasty, replacement, knee, knee joint, knee prosthesis, prosthesis design

## Abstract

**Objetivo:**

Este estudo comparou os desfechos clínicos, a sobrevida do implante e a mobilidade axial entre próteses de plataforma móvel (PM) e de plataforma fixa (PF) em pacientes com osteoartrite medial do joelho.

**Métodos:**

Foi realizado um estudo de coorte retrospectivo de 1.289 pacientes submetidos à artroplastia total cimentada primária do joelho (ATJ) entre 2003 e 2022. As próteses PM foram utilizadas em 820 pacientes (seguimento médio de 8,1 anos) e as PF em 469 pacientes (seguimento médio de 15,2 anos). Os desfechos funcionais foram avaliados com os escores International Knee Documentation Committe (IKDC) e Kujala. A amplitude de movimento e a rotação axial da tíbia foram avaliadas clinicamente. Os testes estatísticos incluíram análise de variância, testes *t* e F de Fisher (significância de *p* < 0,05).

**Resultados:**

Ambos os grupos apresentaram melhora funcional significativa ( *p* < 0,001). No seguimento final, não foram encontradas diferenças significativas entre PM e PF nos escores IKDC ou Kujala. A sobrevida do implante foi de 96,3% (PM) *versus* 95,7% (PF) ( *p* = 0,67). A rotação tibial axial foi significativamente maior em PM (23,1 ± 4,5°) do que em PF (19,4 ± 4,2°) ( *p* = 0,003). Não ocorreram deslocamentos da plataforma.

**Conclusão:**

Os modelos PM e PF oferecem benefícios funcionais duráveis. Embora a PM tenha proporcionado maior mobilidade axial, ela não resultou em desfechos funcionais superiores ou longevidade do implante. A seleção da prótese deve ser adaptada às necessidades individuais do paciente, à preferência do cirurgião e ao custo. Mais estudos prospectivos são necessários para definir a relevância clínica da cinemática aprimorada.

## Abstract (en)

**Objective:**

The present study compared clinical outcomes, implant survival, and axial mobility between mobile-bearing (MB) and fixed-bearing (FB) prostheses in patients with medial knee osteoarthritis.

**Methods:**

A retrospective cohort study of 1,289 patients who underwent primary cemented total knee arthroplasty (TKA) from 2003 to 2022 was conducted. Mobile-bearing prostheses were used in 820 patients (mean follow-up: 8.1 years), and FB in 469 patients (mean follow-up: 15.2 years). Functional outcomes were assessed using the International Knee Documentation Committee (IKDC) and Kujala scores. Range of motion and axial tibial rotation were clinically evaluated. Statistical tests included analysis of variance, *t* -tests, and Fisher's F-test (significance *p* < 0.05).

**Results:**

Both groups showed significant functional improvement ( *p* < 0.001). At the final follow-up, no significant differences were found between MB and FB in the IKDC or Kujala scores. Implant survival was 96.3% (MB) versus 95.7% (FB) ( *p* = 0.67). Axial tibial rotation was significantly higher in MB (23.1 ± 4.5°) than in FB (19.4 ± 4.2°) ( *p* = 0.003). No bearing dislocations occurred.

**Conclusion:**

Mobile-bearing and FB designs offer durable functional benefits. Although MB provided greater axial mobility, it did not result in superior functional outcomes or implant longevity. Prosthesis selection should be tailored to individual patient needs, surgeon preference, and cost. Further prospective studies are needed to define the clinical relevance of enhanced kinematics.

## Introdução

A osteoartrite do joelho (OJ) é uma doença articular degenerativa de origem multifatorial. Resulta do desgaste progressivo e dos danos à cartilagem articular. A OJ, ou gonartrose, é uma condição comum em adultos mais velhos. Sua prevalência continua a aumentar devido ao aumento da expectativa de vida e à obesidade. As estimativas variam dependendo da fonte, com prevalência média de 13% em mulheres e 10% em homens aos 60 anos, aumentando para 40% em pacientes na casa dos 70 anos. 1

O tratamento depende da gravidade dos sintomas e do grau de degeneração articular, classificados segundo o sistema de Kellgren e Lawrence. 2 Opções não cirúrgicas e cirúrgicas estão disponíveis; entre as cirúrgicas está a artroplastia total do joelho (ATJ), que é indicada para casos graves em pacientes altamente sintomáticos. 3

Nos Estados Unidos, a ATJ é o segundo procedimento cirúrgico mais realizado, com um aumento de 134% nos últimos 20 anos. 4

Dois modelos comuns de ATJ são os implantes de plataforma fixa (PF) e de plataforma móvel (PM). Os modelos PF têm um inserto de polietileno fixado à placa de base tibial, enquanto os implantes PM permitem rotação axial ou leve translação anteroposterior. 5 6 Os modelos PM introduzidos na década de 1980 visavam imitar a mecânica fisiológica, reduzir a tensão de cisalhamento, aumentar a conformidade e minimizar o desgaste. 7 8

Biomecanicamente, os modelos de PM podem reduzir o estresse de contato e acomodar desalinhamentos cirúrgicos, potencialmente preservando a reversão e a rotação tibial durante a flexão. 9 10 11 Alguns estudos cinemáticos mostram melhor rotação axial com a PM, mas sem benefícios clínicos consistentes. Os implantes de PM também apresentam riscos, como deslocamento do inserto, aumento da complexidade cirúrgica e maior custo. 12

Dados de longo prazo são cruciais. Enquanto revisões anteriores sugeriram pequenas vantagens da PM, estudos recentes com seguimentos mais longos mostram resultados mistos. 13

Não há consenso sobre se os implantes de PM oferecem longevidade superior, melhor biomecânica ou melhores resultados para os pacientes.

Este estudo teve como objetivo comparar os resultados a longo prazo de ATJ com PM e PF ao longo de 15 e 20 anos. Avaliamos as taxas de sobrevida, a função e a revisão do implante, hipotetizando que não há diferenças significativas na longevidade, na função ou nas complicações da prótese entre os dois modelos.

## Materiais e Métodos

O estudo foi aprovado pelo nosso comitê de ética em 20 de janeiro de 2025.

### Desenho do Estudo e da População

Este estudo retrospectivo unicêntrico incluiu 1.289 pacientes submetidos à ATJ para osteoartrite do compartimento medial entre 2003 e 2022; todos os pacientes foram operados pela mesma equipe ortopédica em um centro acadêmico de alto volume. Os pacientes foram agrupados por tipo de implante: 820 receberam próteses de PM (seguimento médio de 8,1 anos) e 469 receberam próteses de PF (seguimento médio de 15,2 anos). Todos apresentavam alinhamento em varo (ângulo quadril-joelho-tornozelo < 180°) e osteoartrite medial e femoropatelar, com falha no tratamento conservador.

Os critérios de inclusão foram graus Kellgren-Lawrence ≥ III, ATJ cimentada primária (PM ou PF) e ≥ 2 anos de seguimento. Os critérios de exclusão incluíram artrite inflamatória, osteotomia ou trauma prévio, deformidade grave (> 15° varo/valgo ou > 20° contratura), perda óssea importante, cirurgia de revisão ou dados de seguimento incompletos.

### Técnica Cirúrgica

Todas as cirurgias seguiram um protocolo padronizado utilizando uma abordagem parapatelar medial 14 com torniquete. A cefazolina (2 g) foi administrada 30 minutos antes da incisão. Ambos os ligamentos cruzados foram ressecados. Os cortes ósseos seguiram a modelagem pré-operatória; a rotação femoral foi ajustada em 3° a 5° para rastreamento patelar. Alinhamento mecânico visando um eixo neutro. Os componentes de teste garantiram equilíbrio e estabilidade adequados. Todos os componentes foram cimentados com pressurização; as patelas receberam o componente patelar. Não foram utilizados drenos. No pós-operatório, os pacientes receberam analgesia multimodal, iniciaram a mobilização às 24 horas e foram submetidos à tromboprofilaxia com enoxaparina. A medicação de alta incluiu analgésicos (acetaminofeno e antiinflamatórios não esteroides [AINEs]), relaxantes musculares e proteção gástrica por 10 dias, além de tromboprofilaxia com anticoagulantes orais diretos (rivaroxabana 10 mg) por 30 dias. Todos seguiram um protocolo de reabilitação padronizado com carga progressiva.

### Avaliação do Resultado Funcional

Os desfechos clínicos foram avaliados com duas medidas validadas relatadas pelo paciente: o escore subjetivo do International Knee Documentation Committee (IKDC) e a Escala de Dor Anterior no Joelho de Kujala. As avaliações foram realizadas aos 1, 3, 6 e 12 meses e aos 3, 5, 10, 15 e 20 anos de pós-operatório. O escore IKDC (0–100) avalia sintomas, função e desempenho do joelho; escores mais altos indicam melhores resultados. 15 A escala de Kujala avalia a função femoropatelar, incluindo dor, claudicação e instabilidade, e escores mais altos indicam melhor função. 16 A amplitude de movimento de flexão-extensão foi medida com um goniômetro. A rotação tibial axial foi avaliada clinicamente em 90° de flexão, por meio da escala visual analógica (EVA).

### Análise Estatística

A análise estatística foi realizada no software R (R Foundation for Statistical Computing). A estatística descritiva sintetizou as variáveis demográficas e clínicas. A análise de variância ( *analysis of variance* , ANOVA, em inglês) unidirecional ou testes t não pareados compararam variáveis contínuas, enquanto testes t pareados avaliaram a melhora funcional no grupo. A análise de Kaplan-Meier avaliou a sobrevida do implante. Para reduzir o viés decorrente de durações de seguimento desiguais, a análise de sensibilidade encurtou o seguimento para 10 anos. A regressão linear multivariada examinou o efeito independentemente do tipo de implante nos escores IKDC e Kujala, ajustando por idade, sexo, índice de massa corporal (IMC), alinhamento e seguimento. A correspondência do escore de propensão (vizinho mais próximo 1:1; margem = 0,2 desvio padrão [DP]) criou coortes equilibradas. Os modelos de riscos proporcionais de Cox avaliaram a sobrevida livre de revisão, ajustando para as mesmas covariáveis. As suposições de riscos proporcionais foram verificadas por meio de resíduos de Schoenfeld e métodos gráficos. A significância foi estabelecida em *p* < 0,05.

## Resultados

### Características Basais

Um total de 1.289 pacientes que preencheram os critérios de inclusão foi analisado: 820 no grupo PM e 469 no grupo PF. As duas coortes foram estatisticamente comparáveis no início do estudo. A idade média na cirurgia foi de 68,3 ± 7,2 anos no grupo PM e 68,6 ± 6,8 anos no grupo PF ( *p* = 0,41), e a proporção de mulheres foi semelhante (72,4% *versus* 68,0%, respectivamente; *p* = 0,09). A distribuição de lateralidade (procedimentos à esquerda, à direita ou bilaterais) e os graus de osteoartrite de Kellgren-Lawrence também não mostraram diferenças significativas ( *p* > 0,05 em todas as comparações). A ANOVA unidirecional confirmou a homogeneidade da distribuição etária (F = 1,27; F-crítico = 3,84; *p* = 0,26), o que apoiou a comparabilidade de ambas as coortes.

### Desfechos Funcionais: Melhoria Intragrupo

Ambos os modelos de implantes demonstraram melhora funcional significativa ao longo do tempo. No grupo PF, o escore médio do IKDC aumentou de 45,2 no pré-operatório para 84,9 aos 20 anos. Em paralelo, a pontuação de Kujala melhorou de 48,1 para 90,0. No grupo PM, o escore do IKDC melhorou de 46,1 no período basal para 90,0 aos 15 anos e o escore do Kujala de 47,6 para 90,1 ( Tabela 1 ).

**Tabela 1:** Média e desvio padrão dos escores do IKDC e Kujala ao longo do tempo (ATJ com PF *versus* PM)

Tempo	IKDC PF (média ± DP)	IKDC PM (média ± DP)	Kujala PF (média ± DP)	Kujala PM (média ± DP)
**Pré-operatório**	45,2 ± 3,1	46,1 ± 3,2	48,1 ± 3,1	47,6 ± 3,2
**1 mês**	52,5 ± 4,7	52,5 ± 4,5	52,5 ± 4,8	52,7 ± 4,6
**3 meses**	67,4 ± 4,5	67,6 ± 4,7	67,7 ± 4,6	67,5 ± 4,5
**6 meses**	77,2 ± 3,2	75,1 ± 3,2	75,4 ± 3,2	75,2 ± 3,2
**1 ano**	80,2 ± 3,2	80,1 ± 3,1	79,9 ± 3,0	79,9 ± 3,2
**3 anos**	85,1 ± 3,1	84,9 ± 3,2	84,9 ± 3,2	84,8 ± 3,2
**5 anos**	85,2 ± 3,1	85,1 ± 3,1	84,8 ± 3,2	85,1 ± 3,3
**10 anos**	90,2 ± 3,1	90,0 ± 3,2	89,9 ± 3,2	90,0 ± 3,2
**15 anos**	87,5 ± 1,7	90,0 ± 3,1	90,0 ± 3,2	90,1 ± 3,2
**20 anos**	84,9 ± 3,1	—	90,0 ± 3,1	—

Abreviaturas: ATJ, artroplastia total do joelho; DP, desvio padrão; IKDC, International Knee Documentation Committe; PF, plataforma fixa; PM, plataforma móvel.

Os testes *t* pareados mostraram que essas melhorias foram estatisticamente significativas em ambos os grupos, em todos os momentos ( *p* < 0,001), refletindo trajetórias de recuperação intragrupo robustas ( Tabela 2 ).

**Tabela 2:** Comparação entre os grupos: pré-operatório *versus* seguimento final

Escore	Grupo	Pré-operatório (média ± DP)	Seguimento final	Média Δ	Valor de *p* (teste *t* pareado)
**IKDC**	PF	45,2 ± 3,1	84,9 ± 3,1	+39,7	< 0,001
**IKDC**	PM	46,1 ± 3,2	88,3 ± 3,1	+42,2	< 0,001
**Kujala**	PF	48,1 ± 3,1	89,9 ± 3,2	+41,8	< 0,001
**Kujala**	PM	47,6 ± 3,2	90,0 ± 3,2	+42,4	< 0,001

Abreviaturas: DP, desvio padrão; IKDC, International Knee Documentation Committe; PF, plataforma fixa; PM, plataforma móvel.

### Comparação Funcional Intergrupo

Ao longo do seguimento, os escores IKDC e Kujala foram consistentemente maiores no grupo PM do que no PF, embora as diferenças não tenham sido estatisticamente significativas ( *p* > 0,05). A ANOVA de medidas repetidas não mostrou interação significativa entre o tipo de implante e a progressão do escore ( *p* = 0,18), indicando trajetórias funcionais semelhantes. No entanto, a melhora média do IKDC foi de 42,2 pontos em PM *versus* 39,7 em PF, e a melhora de Kujala foi de 42,4 pontos *versus* 41,8, sugerindo uma tendência não significativa a favor dos implantes de PM nos resultados relatados pelo paciente ( Tabela 3 ).

**Tabela 3:** Comparação entre os grupos da melhora funcional (Δ pré-operatório até o seguimento final)

Escore	Δ PF (média ± DP)	Δ PM (média ± DP)	Diferença média	Valor de *p* (teste *t* independente)
**IKDC**	39,7 ± 3,4	42,2 ± 3,5	+2,5	0,08
**Kujala**	41,8 ± 3,5	42,4 ± 3,6	+0,6	0,41

Abreviaturas: DP, desvio padrão; IKDC, International Knee Documentation Committe; PF, plataforma fixa; PM, plataforma móvel.

Após a correspondência do escore de propensão, 392 pares correspondentes foram identificados com características de linha basal equilibradas. Nesta coorte pareada, nenhuma diferença significativa foi observada no IKDC final (PM: 87,6 ± 8,2 *versus* PF: 86,3 ± 8,5, *p* = 0,21) ou nos escores de Kujala (PM: 84,7 ± 7,6 *versus* PF: 82,9 ± 8,2, *p* = 0,18), o que espelha os resultados da população não pareada.

No modelo de regressão multivariável, o modelo de PM não foi um preditor independente de IKDC (β = 1,23; IC95%: −0,85–3,31; *p* = 0,24) ou de escores de Kujala melhorados (β = 1,46; IC95%: −0,74–3,67; *p* = 0,19) após o ajuste por fatores de confusão. No entanto, os implantes PM permaneceram significativamente associados a uma rotação axial maior da tíbia (β = 3,4°; IC95%: 1,7–5,1°; *p* < 0,001).

### Amplitude de Movimento e Mobilidade Rotacional

A amplitude de movimento (ADM) pós-operatória foi comparável entre os grupos. Aos 15 anos, a flexão média foi de 122,7 ± 6,8° no grupo PM e de 121,9 ± 7,1° no grupo PF ( *p* = 0,21). Os déficits de extensão foram mínimos em ambos os grupos (−1,3 ± 1,1° *versus* −1,4 ± 1,0°; *p* = 0,48), sem diferença estatisticamente significativa no arco de flexo-extensão. É importante ressaltar que a rotação axial tibial, avaliada clinicamente a 90° de flexão, revelou diferença significativa entre as coortes: o grupo PM exibiu um arco de rotação total médio de 23,1 ± 4,5°, em comparação com 19,4 ± 4,2° no grupo PF ( *p* = 0,003). Isso sugere preservação superior do movimento rotacional fisiológico nos implantes de PM, alinhando-se com sua vantagem teórica de projeto cinemático ( Tabela 4 ).

**Tabela 4:** Amplitude de movimentos e rotação axial aos 15 anos de seguimento

Variável	Grupo PF (média ± DP)	Grupo PM (médiaDP)	Valor de *p*
**Flexão máxima do joelho (°)**	121,9 ± 7,1	122,7 ± 6,8	0,21
**Déficit de extensão (°)**	−1,4 ± 1,0	−1,3 ± 1,1	0,48
**Rotação tibial axial (°)**	19,4 ± 4,2	23,1 ± 4,5	0,003

Abreviaturas: DP, desvio padrão; PF, plataforma fixa; PM, plataforma móvel.

### Sobrevida e complicações do implante

A sobrevida livre de revisão cumulativa no seguimento final foi de 96,3% no grupo PM e de 95,7% no grupo PF. A diferença não foi estatisticamente significativa (teste de log-rank, *p* = 0,67). As causas de revisão incluíram afrouxamento asséptico (n = 4 PM; n = 5 PF), desgaste de polietileno (n = 3 PM; n = 2 PF) e complicações de rastreamento patelar (n = 2 PF). Nenhum caso de luxação da plataforma foi relatado no grupo PM, e nenhuma infecção profunda ocorreu em ambos os grupos.

**Fig. 1:**
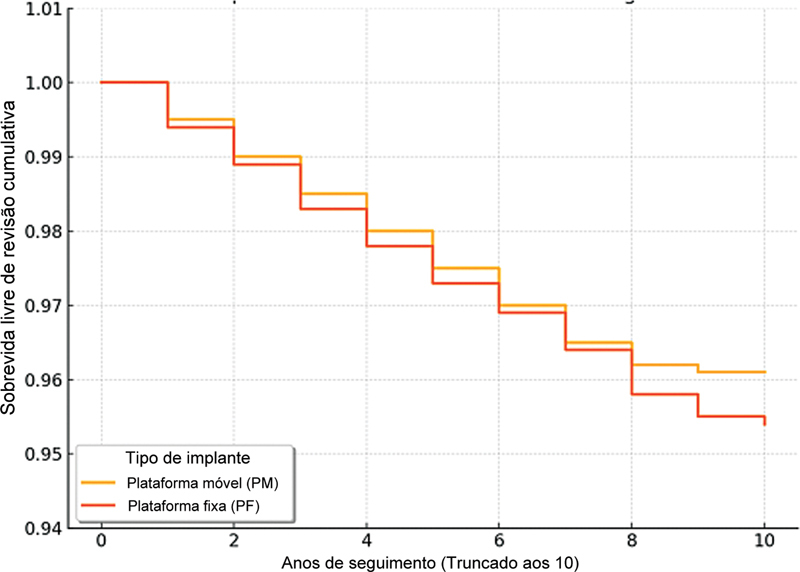
Curvas de sobrevida (Kaplan–Meier).

Ao encurtamento do seguimento aos 10 anos para explicar as diferenças no tempo de observação, a sobrevida livre de revisão cumulativa permaneceu elevada em ambos os grupos: 96,1% para PM e 95,4% para PF (teste de log-rank, *p* = 0,72). As curvas de Kaplan-Meier mostraram intervalos de confiança sobrepostos, confirmando a estabilidade da tendência de sobrevida ao longo do tempo ( Fig. 1 ).

### Regressão Multivariável e Análise de Sobrevida Ajustada

No modelo de regressão linear multivariável para o escore IKDC, o implante de PM não foi associado, independentemente, a melhores desfechos (β = 1,17; IC95%: −0,73–3,07; *p* = 0,23). Da mesma forma, no modelo de escore de Kujala, a associação permaneceu estatisticamente não significativa (β = 1,32; IC95%: −0,62–3,26; *p* = 0,18). Essas descobertas confirmam que as diferenças de pontuação bruta entre os grupos foram amplamente atribuídas às variações da linha basal e à duração do seguimento, e não ao próprio tipo de implante.

Notavelmente, a rotação tibial axial foi um preditor independente significativo do escore IKDC (β = 0,51 por aumento de grau; IC95%: 0,19–0,83; *p* = 0,002), indicando que a preservação do movimento rotacional pode ter relevância funcional além do projeto do implante por si só.

No modelo de riscos proporcionais de Cox, ajustado por idade, sexo, IMC, alinhamento e tempo de seguimento, a razão de risco (HR) para revisão no grupo PM, em comparação com o PF, foi de 0,94 (IC95%: 0,62–1,41; *p* = 0,76), sugerindo nenhuma diferença significativa na sobrevida do implante após o controle de fatores de confusão. Idade, sexo e alinhamento não foram significativamente associados ao risco de revisão, embora o IMC tenha apresentado associação limítrofe (HR = 1,03 por unidade; IC95%: 0,99–1,07; *p* = 0,08).

### Análise de Subgrupo e Correlação entre Rotação Axial e Função

As análises de subgrupos foram conduzidas por idade (< 65 *versus* ≥ 65 anos), sexo, IMC (< 30 *versus* ≥ 30 kg/m ^2^ ) e função basal (IKDC < 50 *versus* ≥ 50) para avaliar o impacto clínico dos implantes de PM. Em pacientes < 65 anos, os implantes de PM apresentaram IKDC final ligeiramente maior (89,4 ± 7,6 *versus* 86,7 ± 8,3; *p* = 0,048) e escores de Kujala (86,2 ± 6,9 *versus* 83,1 ± 7,4; *p* = 0,044) do que os implantes de PF. As diferenças não foram significativas em pacientes ≥ 65 anos ( *p* > 0,1). Não foram observadas diferenças significativas por sexo ou IMC.

Em pacientes com melhor função pré-operatória (IKDC ≥ 50), os implantes PM resultaram em maior rotação tibial axial (24,5 ± 4,1° *versus* . 20,1 ± 4,4°; *p* < 0,001) e maiores escores de Kujala (85,4 ± 7,1 *versus* 82,5 ± 7,6; *p* = 0,035). Esses efeitos não foram significativos naqueles com IKDC < 50. A análise de correlação mostrou associações moderadas em pacientes mais jovens (r = 0,34; *p* < 0,01) e em pacientes de alto funcionamento (r = 0,29; *p* = 0,016), mas não em geral (r = 0,11; *p* = 0,14).

### Testes de Hipóteses e Validação Estatística

O teste F de Fisher, comparando a variância dos escores IKDC e Kujala de longo prazo entre os grupos PM e PF, produziu um valor F de 1,18, abaixo do limiar crítico de 3,84 ( *p* = 0,28). Assim, a hipótese nula—afirmando que não há diferença significativa nos desfechos funcionais entre os dois tipos de prótese—não poderia ser rejeitada.

No entanto, a análise de subgrupos post hoc revelou uma vantagem estatisticamente significativa do modelo PM na preservação da capacidade rotacional tibial, conforme discutido acima. Embora os desfechos funcionais gerais tenham sido estatisticamente equivalentes, essa vantagem biomecânica específica pode ter relevância clínica em pacientes mais jovens ou mais ativos.

## Discussão

O principal achado deste estudo retrospectivo é que os modelos de ATJ com PM e PF proporcionaram melhorias funcionais significativas e duráveis em pacientes com osteoartrite do compartimento medial. Não foram observadas diferenças estatisticamente significativas nas medidas de resultado relatadas pelo paciente (PROMs), incluindo os escores IKDC e Kujala. No entanto, os implantes PM demonstraram maior rotação tibial axial no seguimento a longo prazo (23,1° *versus* 19,4°; *p* = 0,003), sugerindo uma potencial vantagem cinemática.

Implantes PM permitem movimento relativo entre o inserto de polietileno e a placa de base tibial, permitindo rotação axial controlada e translação limitada. Essas características podem reduzir as forças de cisalhamento e replicar melhor o movimento natural do joelho. Nossos resultados são consistentes com estudos cinemáticos anteriores: Fransen et al., 17 relataram melhor controle rotacional e maior flexão com implantes PM durante a marcha. Hanusch et al., 18 Harrington et al. 19 e Hasegawa et al. 20 encontraram maior rotação tibial e mobilidade axial em condições dinâmicas.

Apesar desses benefícios biomecânicos, a relevância clínica permanece debatida. Revisões sistemáticas e metanálises mostraram que os implantes PM não superam consistentemente o PF em escores funcionais, sobrevida ou taxas de complicações. 20 21 Nossos achados apoiam isso: a melhoria da mobilidade rotacional não se traduziu em melhores resultados gerais ao longo de até 20 anos de seguimento.

Este estudo acrescenta novas evidências ao avaliar a rotação axial, raramente estudada em grandes coortes. Em pacientes mais jovens ou altamente ativos, os implantes PM podem oferecer benefícios modestos, porém significativos. No entanto, o pequeno ganho rotacional, combinado com PROMs e sobrevida semelhantes, não justifica o uso rotineiro de PM em todos os casos de ATJ.

Em uma meta-análise, Hantouly et al., 22 em 2021 de estudos randomizados comparando ATJ com implantes de PM versus PF com um seguimento de ≥ 12 meses foram incluídos. Não foram identificadas diferenças nas taxas de revisão, afrouxamento, escores funcionais, amplitude de movimento ou achados radiográficos. Em conclusão, ambos os modelos alcançaram excelentes resultados, e as vantagens teóricas do inserto de plataforma móvel não foram confirmadas. 22

Em 2022, um estudo prospectivo, randomizado e controlado de Sohn et al. 23 comparou 49 ATJs de PF com 49 PM, avaliando a consciência articular e a crepitação, bem como a amplitude de movimento, os escores funcionais, a posição do implante e o nível da linha articular. Os resultados não mostraram diferenças significativas entre os grupos na Pontuação Conjunta Esquecida, na incidência ou na gravidade da crepitação, na amplitude de movimento, nas pontuações funcionais ou nos resultados radiográficos. Eles concluíram que a ATJ com PM não mostrou benefícios sobre a ATJ com PF; as vantagens teóricas do inserto da plataforma móvel não foram confirmadas, deixando a escolha do implante para o cirurgião. 23

Finalmente, o estudo mais recente, realizado em 2024 por Kim et al., 24 comparou 88 pacientes, com idade média de 66 anos, que receberam PM ou PF, avaliados clinicamente (EVA, ADM, Knee Society Score [KSS] e Western Ontario and McMaster Universities Osteoarthritis Index [WOMAC]) e radiograficamente aos 13 anos de seguimento. Não foram encontradas diferenças significativas entre os grupos PM e PF nos desfechos clínicos ou radiográficos, nem na incidência de complicações ( *p* > 0,05). Eles concluíram que, embora os desfechos clínicos e radiográficos fossem semelhantes, o risco potencialmente maior de osteólise ou afrouxamento asséptico na ATJ PM poderia influenciar a escolha do implante.

## Implicações Clínicas

A seleção do implante deve ser individualizada com base em fatores como idade, atividade, alinhamento e equilíbrio dos tecidos moles. Os implantes de PM podem beneficiar pacientes que precisam de mobilidade axial aprimorada, enquanto os implantes de PF permanecem confiáveis e econômicos na maioria dos casos de ATJ. Análises secundárias—seguimento truncado, ajuste multivariável e correspondência de propensão—não confirmaram diferenças significativas na função ou na sobrevida a longo prazo. A rotação axial manteve-se consistentemente maior no grupo PM, reforçando sua relevância biomecânica. Embora o implante de PM não tenha sido um preditor independente de resultados, a maior rotação tibial correlacionou-se com escores mais elevados de IKDC, destacando o papel da cinemática articular na otimização dos resultados.

## Limitações

Este estudo apresenta algumas limitações. Primeiro, a duração do seguimento diferiu entre as coortes, o que pode ter influenciado as comparações. Embora tenha sido abordado por meio de análise de Kaplan-Meier truncada e de modelos ajustados, o viés residual pode persistir. Em segundo lugar, o design retrospectivo e não randomizado introduz confusão. A pontuação de propensão que corresponde a grupos equilibrados, mas com variáveis não medidas, permanece possível. Em terceiro lugar, as medidas de resultado (escores IKDC e Kujala), embora validadas, são menos comuns na pesquisa de artroplastia, o que limita a comparabilidade com estudos que utilizam Oxford Knee Score (OKS) ou KSS. Além disso, a rotação tibial axial foi avaliada clinicamente, e não por meio de imagem dinâmica, o que reduz a precisão da medição. Pesquisas com análise de movimento ou rastreamento radioestereométrico podem aumentar a precisão. Finalmente, variáveis como o nível de atividade, a adesão à reabilitação e detalhes da técnica cirúrgica não foram capturadas e podem ter influenciado os resultados, apesar do ajuste multivariável.

## Conclusão

Neste estudo retrospectivo de longo prazo de ATJ com PM vs. PF para osteoartrite medial, ambos os implantes mostraram melhora funcional significativa e duradoura. Os implantes de PM demonstraram maior rotação tibial axial, indicando potenciais vantagens cinemáticas, mas não foram observadas diferenças significativas nos resultados relatados pelo paciente nem na sobrevida dos implantes. Esses resultados não sustentam o uso rotineiro de PM sobre PF na ATJ primária. A seleção do implante deve ser baseada na atividade do paciente, nas expectativas, no custo e na experiência cirúrgica. Mais estudos prospectivos são necessários para esclarecer se a mobilidade axial melhorada oferece benefícios clínicos significativos a longo prazo.
